# Dr. Stevens on the Blood in Fever

**Published:** 1830-07-01

**Authors:** 


### OBSERVATIONS ON THE BLOOD. By WILLIAM STEEVENS, M.D.

At a late meeting of the College of Physicians, a paper on the above subject was read by Dr. Steevens, who has, for many years, been a practising physician in the West Indies. As the views which Dr. S. entertains are novel, and the means which he proposes for combating a dreadful scourge of the human race, are simple and practicable, we have endeavoured to collect the substance of the writer's observation as accurately as possible, to lay before our readers.

Dr. S. sets out with remarking that a malignant form of yellow fever is sometimes seen in the West Indies, which, from begining to end, evinces, by the symptoms, little or no affection of the solids, and leaving, after death, no cognizable trace of disease in the brain, stomach, intestines, or other viscus, whose derangements are supposed to be the cause of fever. In those fatal cases, there is no excitement, in the commencement, adequate to the injury of organs, and we can only ascertain the real cause of death when we come to open the heart, and find in its cavities, a dissolved fluid almost as thin as water, and black as ink—a fluid evidently incapable of stimulating the central organ of the circulation or of supporting life. The fluid is equally black and thin throughout the vascular system, all distinction between arterial and venous blood being obliterated.

An attentive observation of some cases of fever first led the author to believe that, in this disease, the influence of the nervous system is less operative than is generally supposed, and that the blood is infinitely more concerned than the solids, in an etiological point of view. He was thus led on, not only to attentive observation, but to experiments on the state of the blood in fever, some of which he has now submitted to the profession. The writer remarks that it has been fashionable, for more than a century, to overlook the pathology of the fluids; yet to the practical physician, the morbid changes which the blood undergoes is valuable beyond all calculation.

On examining, after death, the black and dissolved blood that has been taken from the hearts of those who have died of the yellow fever, it was evident that great changes had taken place. *First*, the blood was more fluid, partly from an excess of serum, probably produced by a stoppage of all the secretions—partly, perhaps, from the non-formation of fibrine in fever, or its more rapid exhaustion than in health. In the first stage of the disease, the structure of the red globules is frequently deranged, as is evident from the colouring matter being often detached from the globules, and dissolved in the serum, giving to that part of the blood, when it separates from the fibrine, a bright scarlet colour. As the disease advances, this red colour is lost, and the whole circulating mass becomes black and thin. *Secondly*, in reference to the change of colour in the blood, both venous and arterial, Dr. S. has frequently filled one glass with the black fluid taken from the heart, and another with the black vomit taken from the stomach—they were so similar that it was almost impossible to distinguish the one from the other. *Thirdly*, in violent continued fevers, the saline matter, like the fibrine, appears to be exhausted faster than it enters the circulation. The blood soon loses a great portion of its saline impregnation, and the entire of its saline taste, the cause of which, the author afterwards endeavours to shew, is owing to the loss or great diminution of the saline matters. *Fourthly*, the blood, though dissolved, was not putrid—the latter state being, indeed, incompatible with life. But dissolution he considers as the first step towards putrescency—and the cause rather than the effect of death; because he has seen the blood, before death, so black and so thin, that it could scarcely be retained within the vessels—oozing from the tongue, the eyes, the skin, and other surfaces.

Conceiving, then, that this dissolved state of the blood was the cause of death, the great object of inquiry was to find out an agent capable of preventing this fatal change. In all climates, Dr. S. observed, the waters of the deep were preserved from putrefaction—and this preservation, he thinks, is probably owing to their saline impregnation. It is also well known that some of the saline medicines possess great antiseptic powers. Saline matters are invariably found in healthy blood, and as these were invariably lost or greatly diminished in the blood of fever patients, Dr. S. was naturally induced to try the effect of saline medicines, in preventing the bad symptoms of tropical fevers. The result of experience, he avers, was a conviction that those agents had, when used at a proper time, a specific effect in preventing the dissolution of the blood. In all the cases where they were timely and properly used, they prevented the fetor of the breath, the stoppage of the secretions, the yellow colour of the skin, the black vomit, and the other fatal symptoms, so common in those cases where these medicines were not administered.

Our author observes that one common property of neutral salts is that of giving a rich arterial colour to venous blood. This property is common to them all, and the degree to which they possess it is, perhaps, the best test of their purity as saline agents. To ascertain the effects of different agents on the blood, he made a number of experiments, in which it was observed—

1st. That all the acids give a dark colour to healthy blood, and in proportion to their strength, change it from red to black, as certainly as they change vegetable colours from blue to red. Even the vegetable acids so completely blackened the blood, that the addition of a little water converted the whole into fluid exactly resembling the black vomit. *Secondly*, the pure alkalis have a similar effect with the acids, in changing the blood from red to black, though not in the same degree. *Thirdly*, the neutral salts immediately changed the venous blood from a dark modena red, to a bright arterial colour. Even those salts that contain a slight excess of alkali, the sub-carbonate of soda for example, immediately give to venous blood a beautiful bright arterial colour. The effects of these experiments are best seen when made on healthy blood. The agents ought first to be dissolved in a little soft water, and then well mixed with the warm blood, before it begins to coagulate.

4thly. When the neutral salts are mixed with the dark and dissolved blood that had been taken from the hearts of those who had died of yellow fever, even the black and dissolved fluid was instantly converted from a black to a bright arterial colour.

The nature of this paper (said Dr. S.) prevents me from entering minutely on the important effects which this saline impregnation produces in the vital fluid; but, in a work, which will soon be published, I shall endeavour to prove, first, that the blood owes its red colour to this saline impregnation. Black appears to be the natural colour of the colouring matter; for, when we take a clot of blood, and deprive it completely of its saline matter, by immersing it in fresh water, the colouring matter soon becomes so black, that even oxygen has no effect in changing its colour. But, when we immerse this black clot in an artificial serum made by dissolving some saline matter in water, the black clot in this clear fluid assumes almost immediately a beautiful bright arterial colour. Secondly, that, to this saline the fibrin owes its fluidity: for it remains fluid only while mixed with this saline matter, and becomes solid when the saline matter leaves it to unite with the serum. *Thirdly*, that the change of form which this saline matter undergoes, when the blood changes from arterial to venous, and from venous to arterial, alters its capacity for caloric, and gives it an influence in supporting the temperature of the system. The saline impregnation also adds to the stimulating quality of the blood, and assists, even in a high temperature, in adding to its powers of self-preservation.

In the present state of our knowlegde, both of pathology and animal chemistry, it is, Dr. S. justly observed, best to rest simply on facts. Those which he has endeavoured to establish in his inquiry are partly the following: —1st. That, in violent continued fevers, even where proper means are used to protect the organs, by reducing the excitement, chemical changes often take place in the whole circulating mass; and, in these fevers, such changes are almost always the sole cause of the mortality. In proportion as the disease advances, the blood loses its solid part, and becomes thin. It loses its saline matter, and becomes both black and vapid. It loses its preservative power, and goes fast to decay. It loses its vitality, and soon becomes incapable of stimulating the heart or of supporting life. In the yellow fever, in the African typhus, in the plague, &c. the dissolution of the blood is a common cause of death. The typhus of cold climates is, comparatively speaking, a mild disease; but even in the common typhus, Dr. S. thinks that similar changes take place in the blood, though in a less degree. 2dly. In all cases of bad fevers, the loss of the saline or preservative power appears to be, in every instance, the chief cause of the entire dissolution of the vital fluid. 3dly. Where (says Dr. S.) proper means are used to protect the organs from the increased excitement, during the early stage of the disease, and after the excitement is sufficiently reduced, when proper nourishment is given and certain saline medicines are timely and judiciously used, the bad symptoms are generally prevented. When proper saline medicines are used, they do not fret the stomach, they act on the intestines as much as is necessary, they keep up all the secretions; particularly that of the kidneys, and enough is absorbed to enter the circulation and prevent the dissolution of the blood, and preserve it until the fever abates, and all the danger is past. This I am warranted to state as a fact, inasmuch as the treatment was commenced in the West Indies in 1827, and since then it has stood the test in several hundred cases of the West India fevers, where it has been tried both by myself and others, and with scarcely a single loss, when we were called to the patients within the first twenty-four hours after the attack, and with very few deaths where we were called in previously to the commencement of the fatal symptoms. My friend Dr. George William Stedman, now of St. Thomas's, and others have adopted the same treatment, and the result in their practice has been similar to that which occurred in my own cases.

In August, 1828, at a time when there was a good deal of sickness in the garrison at Trinidad, this practice was adopted in the military hospital of that island, that is to say, they bled freely, and used active purgatives in the commencement, to reduce the excitement, and afterwards the saline medicines were administered until the fever abated, and during the convalescence the quinine was given in large doses. In a communication which I received from Mr. Greatrex, of the Royals, who at that time had charge of the hospital, he states "that the above system has been applied to three hundred and forty cases or thereabouts, including both the remitting and yellow fevers, admitted into the hospital after the fever had existed, variously from six to seventy-two hours antecedently to an application to the hospital, with such success, that during the last seven months not a case has died." This document is dated about seven months after the commencement of this practice. Mr. Greatrex also states, that within that time three men died having the remitting fever, but they had also abscesses in the lungs, and purulent expectoration. As these three cases were complicated with extensive organic disease in the lungs, it is probable they would have been fatal under any treatment. But out of the three hundred and forty cases of essential fever, which had been treated in the manner described, there was not one death in the Royals from the time that this practice had been adopted, and I may add, that in the West Indies, Trinidad is generally considered as one of the most sickly islands.

It can be clearly proved, that in the West India fevers, those patients that are left entirely to themselves, have a much better chance of recovery than those who are treated with emetics, calomel or antimony, opium or acids, and that these remedies instead of being useful, add greatly to the sufferings of the patient; they decidedly increase the very evils that they are meant to relieve, and add greatly to the mortality in hot climates.

Finally, Dr. S. considers it an error to consider fever as entirely a disease of the solids—and still more so, to treat it merely with reference to the mere state of excitement. In respect to yellow fever, he thinks it can only be treated with success, when we reduce, by active measures, the increased excitement, at the commencement, and then prevent, by proper means, those chemical changes in the blood, which are, in reality, the sources of the diseased action in the solids, and the true cause of the mortalily in these dreadful fevers. Convinced by numerous facts, Dr. S. adopted a mode of treatment widely different from that which he had formerly employed, and, in as far as it has yet gone, the use of the Rochelle salts (tartarized soda) carbonate of soda, and other active saline medicines, at a proper period of the disease, has been attended with a train of success to which the mere Solidists can produce no parallel. He is convinced that, if this practice be generally adopted, the mortality from West India fever will be greatly lessened.

As we have no doubt that Dr. Steevens will pursue this interesting inquiry further, and lay the results of his observations before the profession in a more extended form, we shall abstain from any comments on the present occasion. We have laid a very full and faithful account of the paper before our readers, and leave them, for the present, to draw their own conclusions.

